# Causal links between obesity, lipids, adipokines, and cognition: a bidirectional Mendelian-randomization analysis

**DOI:** 10.3389/fendo.2025.1439341

**Published:** 2025-02-10

**Authors:** Meng Gong, Haichao Liu, Zhixiang Liu, Yongshen Wang, Shiyi Qi, Hong Guo, Song Jin

**Affiliations:** ^1^ School of Acupuncture and Tuina, Chengdu University of Traditional Chinese Medicine, Chengdu, China; ^2^ College of Traditional Chinese Medicine, Guangzhou University of Chinese Medicine, Guangzhou, China; ^3^ School of Health Preservation and Rehabilitation, Chengdu University of Traditional Chinese Medicine, Chengdu, China; ^4^ School of Acupuncture, Fujian University of Traditional Chinese Medicine, Fuzhou, China; ^5^ Department of Rehabilitation, Hospital of Chengdu University of Traditional Chinese Medicine, Chengdu, China

**Keywords:** adipokines, bidirectional, cognitive ability, genetic correlation, lipids, Mendelian randomization, obesity

## Abstract

**Background:**

The aim of this study was to explore the genetic level association between obesity, lipids, adipokines, and cognitive ability using bidirectional Mendelian randomization (MR) strategies.

**Methods:**

Summary data for three obesity indicators [body mass index (BMI), body fat percentage (BFP) and waist-hip ratio (WHR)], three lipid indicators [HDL cholesterol (HDL), LDL cholesterol (LDL) and triglycerides (TG)], three adipokines [circulating leptin (LEP), Agouti-related protein (AgRP) and Adiponectin (APDN)], and four cognitive ability indicators [cognitive function (CF), cognitive performance (CP), simple reaction time (SRT) and fluid intelligence score (FIS)] were collected. Bidirectional inverse-variance weighted Mendelian randomization (MR) was employed to evaluate the relationship between adiposity and cognitive function. We employed genetic instruments for adiposity indicators as exposures in one direction, and repeated the analysis in the opposite direction using instruments for cognitive function. Sensitivity analyses were conducted to explore heterogeneity and potential horizontal pleiotropy.

**Results:**

Genetically predicted adiposity showed robust associations with markers of cognitive ability. Higher genetically predicted obesity indicators (such as BMI, BFP and WHR), and lipid and adipokineslevels (such as HDL and AgRP) with reduced cognitive ability indicators (such as CF and CP). In the opposite direction, FIS and SRT may influence BMI and HDL respectively. MR estimates for the effects of cognition ability on all obesity, lipids and adipokines measures indicated worse FIS and SRT were associated with higher BMI and lower HDL.

**Conclusions:**

Our MR reveals that high BMI, BFP, WHR and AgRP have negative causal direct effects with cognitive ability, while high HDL and ADPN have positive causal direct effects with cognitive ability. For the reverse causal direction, our consistent findings that worse cognitive function such as SRT and FIS may influence serum HDL level and BMI.

## Introduction

The increasing prevalence of obesity has accentuated concerns regarding its impact on public health. Obesity is widely acknowledged as a prominent risk factor for numerous ailments, including cardiovascular disease, metabolic syndrome, hypertension, diabetes, etc. (1) Moreover, recent studies have demonstrated the detrimental effects of adiposity on cognitive function (2). Conversely, less known, is the influence of cognition on obesity (3). Consequently, it is imperative to prioritize research that investigates the bidirectional relationship between obesity and cognitive function, thus advancing our understanding of this complex interplay.

Obesity is consistently linked to lower cognitive functioning across various age groups, including adults, middle-aged individuals, older adults, and even young children (4–6). It is notably with poor cognitive flexibility, psychomotor speed, intelligence, attention. While both generalized and visceral obesity have been linked to reduced cognitive performance (7), the impact of adiposity location (i.e. central vs. peripheral) on the adiposity-cognition relationship remains uncertain. This uncertainty arises from the predominant use of body mass index (BMI) as a measure of overall obesity in research studies (8, 9). Recent reports suggest that waist-to-hip ratio (WHR) may independently contribute to adverse cognitive outcomes, regardless of BMI. Additionally, in terms of total body fatness, the association between body fat percentage (BFP) and cognitive ability has also yielded contradictory evidence (10, 11).

High-density lipoprotein (HDL), low-density lipoprotein (LDL) cholesterol, and triglycerides (TG) are among the highest commonly measured biomarkers in clinical medicine (12). Epidemiological studies have reported associations between HDL, LDL, TG, and cognitive function (13, 14). However, the existing evidence from these studies is limited in establishing a causal relationship, and inconsistencies exist among various studies (15). Adipokines such as leptin (LEP) and adiponectin (ADPN) have been linked to cognitive function (16). Cognitive flexibility may be modulated by abnormal levels of the appetite-regulating agouti-related protein (AgRP) (17). Nevertheless, conflicting results have been reported in several studies (18, 19). For instance, an early study from the United States found no significant correlation between leptin and cognitive abilities (18), and another study did not find an association between serum leptin levels and cognitive decline (19).

Recent studies have demonstrated that cognitive function is not only a consequence but also a contributor to obesity (20). There is evidence supporting a bidirectional relationship between adiposity and cognitive function exists (21). Furthermore, a vicious cycle has been proposed, where adiposity may lead to cognitive function impairment, which in turn exacerbates further cognitive complications (22). As such, it is crucial to consider this complex bidirectional association in future research (23).

Mendelian randomization (MR), a genetics-based approach in epidemiological research, specifically bidirectional MR, can help elucidate the reciprocal causal pathway between adiposity and cognitive function (24). Recently, Tom and colleagues (8) conducted a bidirectional MR of visual memory (VM) and reaction time (RT) on BMI, BFP and WHR, and vice versa. They observed associations between adiposity on cognitive function likely not causal. Conversely, worse visual memory was causally link to lower adiposity. However, this study primarily focused on VM and RT as indicators of cognitive performance, and the relationship between other cognitive performance indicators and adiposity remains unclear. Conflicting findings have been reported elsewhere, with genetically higher visceral adiposity and raised BMI concentration associated with lower cognition (25). This study, however, may be subject to sample selection bias as it primarily included individuals of Asian ancestry in the Genome-Wide Association Studies (GWASs). Furthermore, there is a dearth of MR studies investigating the potential influence of obesity, lipids, and adipokines on cognitive ability.

In this study, therefore, we aimed to utilize univariable, multivariable and bidirectional MR to examine the causal relationship between obesity (BMI, BFP, WHR), lipids (HDL, LDL, TG) and adipokines (LEP, ADPN, AgRP) with cognitive ability. Our objective was to enhance our understanding of the genetic-level associations between a range of obesity, lipids, adipokines and cognitive ability, thereby providing a more comprehensive and elucidated understanding of causality.

## Methods

### GWAS data sources

Several indicators of obesity, lipid and adipokines were identified as the exposures in the study. The obesity indicators were BMI, BFP and WHR. The lipid indicators were HDL, LDL and TG. The adipokines were LEP, AgRP and ADPN. In addition, Cognitive Function (CF), Cognitive Performance (CP), Simple Reaction Time (SRT) and Fluid Intelligence Score (FIS) were defined as the outcome of this study.

We obtained participant data from various sources, including the UK Biobank (UKB) studies and other large consortia such as the Medical Research Council - Imperial College London (MRC-IEU) (26). The UK Biobank (UKB) is a comprehensive resource that contains genotype data from approximately 500,000 individuals across the UK (27). The summary data for BMI, BFP and FIS were collected from the MRC Integrative Epidemiology Unit at the university of bristol (MRC-IEU). The aggregate data for WHR was obtained from a series of anthropometric traits GWASs involving more than 49,960 individuals of European ancestry (28). The Phenotypes available in UKB included HDL, LDL and TG. For LEP, we utilized summary data from a GWAS that evaluated genomic targets for leptin concentrations in 57,232 individuals of mixed ancestry (29). The summary data for AgRP was obtained from a GWAS that evaluated genomic targets for 90 cardiovascular proteins in 30,931 individuals of European ancestry (30). Genetic instruments for ADPN were obtained from summary statistics of ADIPO-Gen. Moreover, the summary data for CF was collected from a GWAS conducted by the within family consortium. The aggregate datas for CP and SRT were respectively obtained from a series GWASs of educational attainment in 257,841 European individuals (31) and influencing information processing speed (32) using 2,378 individuals of European ancestry.

### Ethics statement

Ethical approval and participant consent were obtained from the respective original studies.

### Genetic instrumental variables

**Figure 1:**
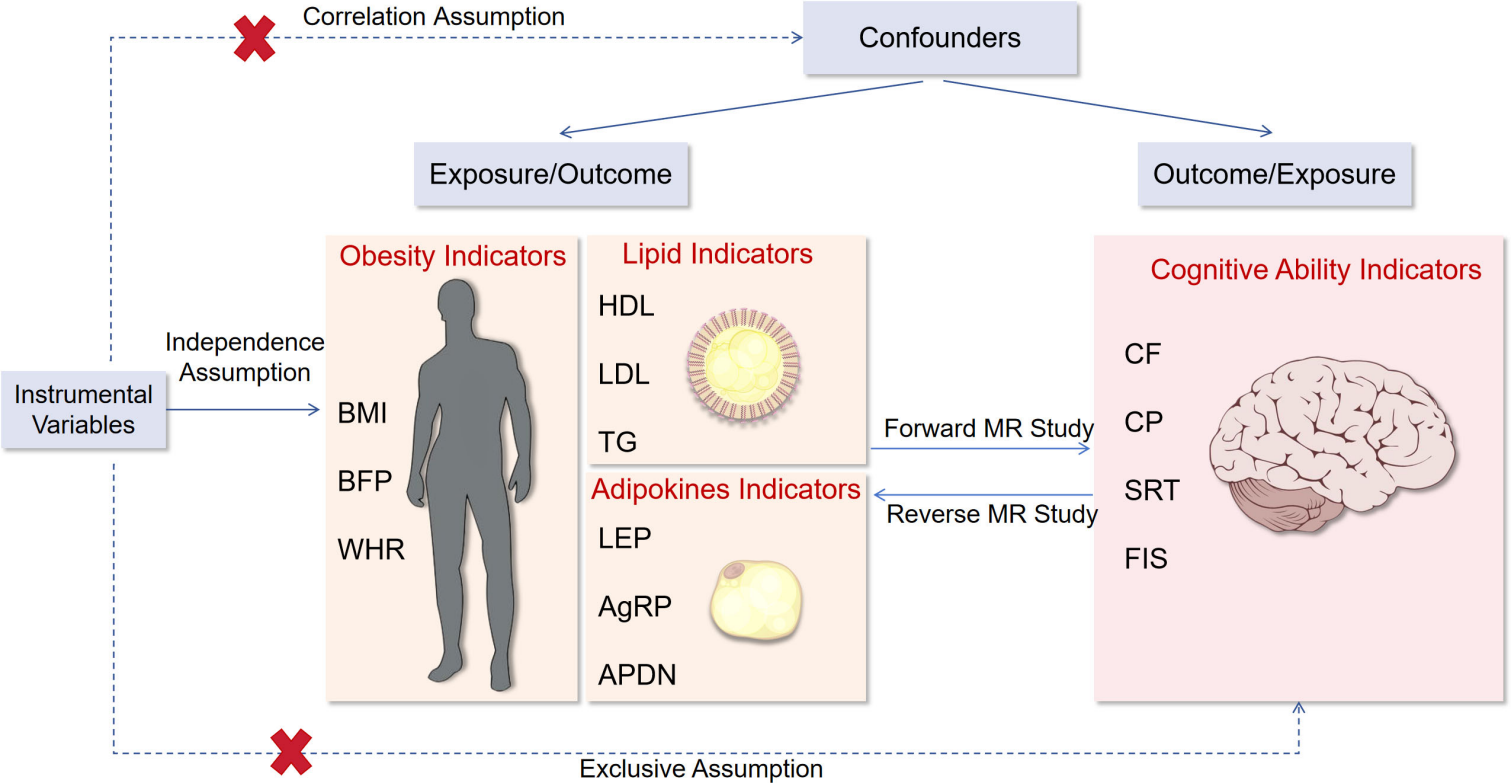
Flow chart of this study. BMI, body mass index; BFP, body fat percentage; WHR, waist-to-hip ratio adjusted for BMI; HDL, high-density lipoprotein cholesterol; LDL, low-density lipoprotein cholesterol; TG, triglycerides; LEP, circulating leptin levels; AgRP, agouti-related protein level; ADPN, adiponectin; CF, cognitive function; CP, Cognitive performance; SRT, simple reaction time; FIS, fluid intelligence score.

From all GWAS summary data of BMI, BFP, etc., we conducted a series of quality control steps to select eligible instrumental SNPs. To meet the correlation assumption, we identified SNPs for inclusion based on two criteria: a genome-wide correlation P-value of less than 5 × 10^−8^ or 5 × 10^−6^ (for SRT), and the absence of linkage disequilibrium (defined as r^2^ > 0.01). To satisfy the independence assumption, we performed the clumping process (r^2^ < 0.001, window size = 10 MB) to estimate linkage disequilibrium between SNPs. Ambiguous SNPs with non-concordant alleles (e.g., A/G vs. A/C) and palindromic SNPs with ambiguous strand (i.e., A/T or G/C) were either corrected or directly excluded from the above-selected instrument SNPs to harmonize the effect of SNPs on the exposure outcome, ensuring that the effects corresponded to the same allele. These rigorously selected SNPs were employed as the instrumental variables in subsequent MR analysis. To ensure that MR analysis meets the three core assumptions. The overall research design is depicted in **Figure 1**.

### Univariable Mendelian randomization

In this two-sample MR, we employed three main methods for causal inference, namely random-effect inverse-weighted variance (IVW), weighted median and MR-Egger (33). The IVW method is considered reasonably accurate for causal inference when all SNPs are unaffected by horizontal pleiotropy and heterogeneity (34). While the other two methods are less accurate, they have broader applicability under certain conditions. Following the harmonization and selection of instrumental variables, we utilized the IVW method to estimate the causal effects of obesity, lipids and adipokines fractions on CF, CP, SRT, FIS.

### Multivariable Mendelian randomization

Multivariable MR is an extension of MR that allows for multiple genetic instruments. In the MVMR method, it is not necessary for the genetic instruments to be exclusively associated with a single risk factor. Instead, they can be associated with a set of measured risk factors, while still satisfying the equivalent instrumental variable assumptions (35). Therefore, we applied this method by considering all instrumental variables for BMI, BFP, WHR, HDL and LDL, TG, as well as LEP, AgRP, and ADPN, respectively, to evaluate their independent effects on CF, CP, SRT, and FIS. Additionally, Reverse-MR analysis was performed to investigate whether CF, CP, SRT, and FIS, as indicators of cognitive ability, may influence obesity, lipid traits, or adipokine traits.

### Reverse Mendelian randomization

Reverse-MR analysis was performed to examine the potential influence of cognitive ability, as represented by CF, CP, SRT and FIS, on obesity, lipids and adipokines traits. Additionally, multivariable MR analysis was also used to assess the causal effect of cognitive ability on obesity, lipids or adipokines traits.

### Sensitivity analyses

Heterogeneity among SNPs was assessed using IVW method and quantified heterogeneities using the Cochran Q statistic. To assess the potential pleiotropic effects of the SNPs used as IVs, we employed the MR-Egger regression. Furthermore, we addressed restriction assumption, by applying method like the MR-PRESSO, which identifies outliers and provides causal estimates after their removal. The MR-PRESSO analysis includes a distortion test that compares estimates before and after outlier removal. We set the number of distributions in MR-PRESSO analysis to 2000. To evaluate the strength of the association between these SNPs and the exposure factors, we used R^2^ and F-statistics to estimate the proportion of phenotypic variance explained and the statistical power, respectively. The F-statistics is calculated using the formula: F = R^2^ × (N − K − 1)/K × (1 − R^2^). We included SNPs with strong statistical power (F statistics > 10) as IVs. Horizontal pleiotropy among SNPs using the MR-Egger intercept test. In all sensitivity analyses, p-values were reported, and the values less than 0.05 were considered indicative of heterogeneity or horizontal pleiotropy. Moreover, we conducted a “leave-one-out” sensitivity analysis to identify potentially influential SNPs. This analysis involved iteratively excluding each SNP one at a time and re-performing the MR analysis to assess the impact of each SNP on the pooled results.

### Statistical analysis

We performed the MR analyses using the two-sample random-effects IVW method, which was implemented in the Mendelian Randomization R package. When there were more tahn 3 SNPs instrumenting the exposures, the estimates for variants were then pooled using the random-effect IVW method. For exposures instrumented by only 2 SNPs, the fixed-effects IVW method was employed. The IVW method was used to access the causal associations along with adiposity and cognitive ability. To account for the documented horizontal pleiotropy between adiposity and cognitive ability, we conducted a multivariable MR (MVMR). Given the high number of comparisons between obesity, lipids and adipokines with cognitive ability (and vice versa) (n = 3 tests for Forward-MR, n = 4 tests for Reverse-MR), we applied a Bonferroni adjustment to all P-value thresholds (i.e. *P*-value threshold/number of tests (3 or 4); *P <*0.05 corresponds to *P <*0.0167, and *P <*0.0125).

The present study adheres to the STROBE-MR guidelines to ensure transparent and thorough reporting of our MR analysis (36). All MR analyses were conducted using TwoSampleMR package in R software (version 4.3.1).

## Results

**Figure 2:**
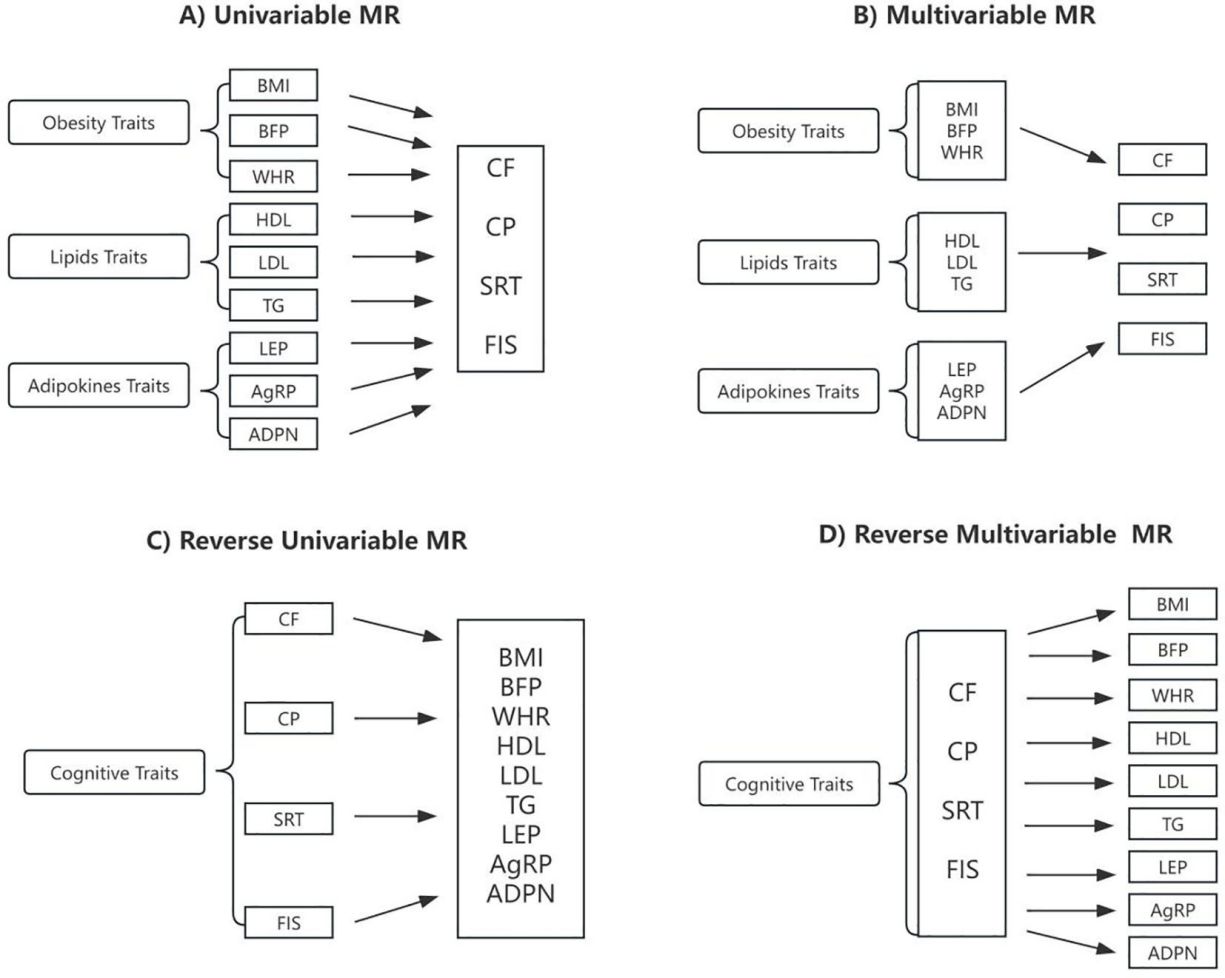
Schematic presentation of **(A)** univariable; **(B)** multivariable; **(C)** reverse univariable; and **(D)** reverse multivariable Mendelian randomization. BMI, body mass index; BFP, body fat percentage; WHR, waist-to-hip ratio adjusted for BMI; HDL, high-density lipoprotein cholesterol; LDL, low-density lipoprotein cholesterol; TG, triglycerides; LEP, circulating leptin levels; AgRP, agouti-related protein level; ADPN, adiponectin; CF, cognitive function; CP, Cognitive performance; SRT, simple reaction time; FIS, fluid intelligence score.

The multivariable and bidirectional MR analysis were conducted according to the workflow presented in **Figure 2**. Details information regarding the instrumental variables selected were listed in **Table 1**.

**Table 1:** Characteristics of the summary data in the study.

T raits (out-come/exposures)	Consortium	Years	Population	Sex	Sample size	F-statistics
Body mass index	MRC-IEU	2018	European	Males and Females	461,460	63.711
Body fat percentage	MRC-IEU	2018	European	Males and Females	454,633	58.263
Waist-to-hip ratio adjusted for BMI	–	2021	European	Males and Females	458,349	79.660
HDL cholesterol	UK Biobank	2020	European	Males and Females	403,943	151.958
LDL cholesterol	UK Biobank	2020	European	Males and Females	440,546	172.155
Triglycerides	UK Biobank	2020	European	Males and Females	441,016	142.067
Circulating leptin levels	–	2020	Mixed	Males and Females	56,802	46.470
Agouti-related protein levels	–	2020	European	Males and Females	21,758	43.185
Adiponectin	ADIPOGen	2012	Mixed	Males and Females	39,883	88.935
Cognitive function	Within family GWAS consortium	2022	European	Males and Females	22,593	31.680
Cognitive performance	–	2018	European	Males and Female	30038396	43.742
Simple reaction time	–	2011	European	Males and Females	2,378	22.356
Fluid intelligence score	MRC-IEU	2018	European	Males and Females	149,051	40.296

### Associations of genetically predicted obesity, lipids and adipokines with cognitive ability

#### Univariable MR analyses

**Figure 3:**
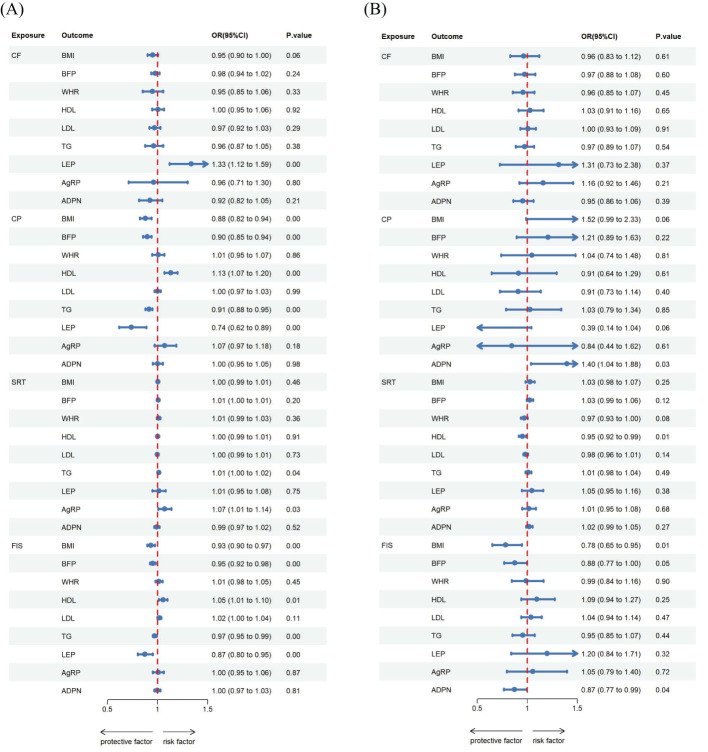
Estimated causal effects of cognitive ability on obesity, lipids and adipokines markers using univariable and multivariable MR, presented as the SD-unit change in obesity, lipids and adipokines marker per genetically predicted 1-SD change in cognitive ability. Schematic presentation of **(A)** univariable; **(B)** multivariable Mendelian randomization.

**Figure 4:**
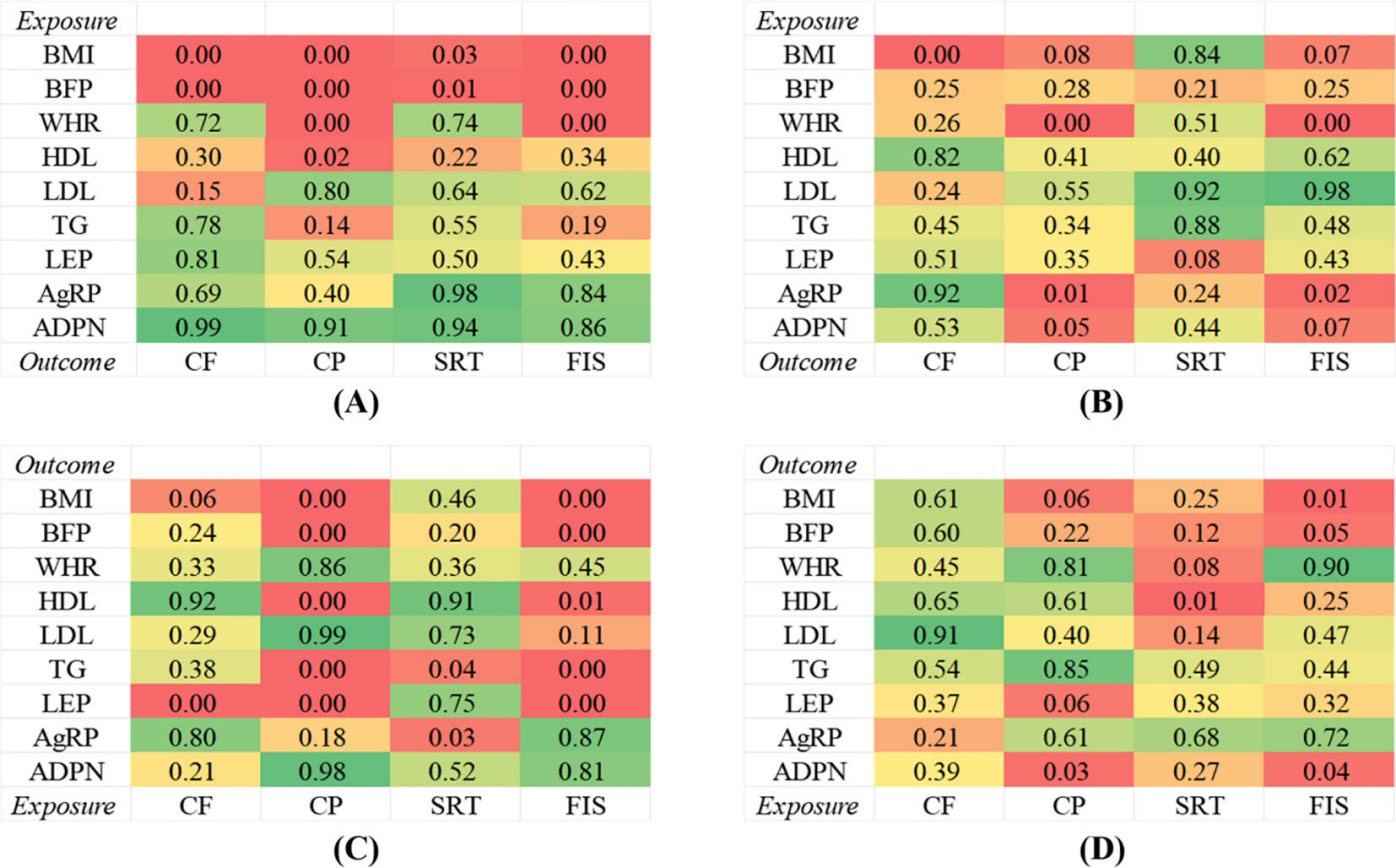
Genetic correlations between obesity, lipids and adipokines factors and cognitive ability. **(A)** Univariable MR between obesity, lipids and adipokines factors on cognitive ability. **(B)** Multivariable MR between obesity, lipids and adipokines factors on cognitive ability. **(C)** Univariable MR between cognitive ability on obesity, lipids and adipokines factors. **(D)** Multivariable MR between cognitive ability on obesity, lipids and adipokines factors.

The relationships between genetically predicted obesity traits and cognitive ability are presented in **Table 2A** and visualized in **Figures 2A**, **3A**, **4A**. We found that the estimated effects (β) of obesity traits of BMI and BFP on CF had the following values within the 95% confidence intervals (CI) were 0.815([0.761 to 0.873]; *P* = 4.948 × 10^-9^), and 0.752([0.680 to 0.833]; *P* = 4.591 × 10^-8^). For SRT, the effect estimates (β) [95% confidence intervals (CIs)] of BMI and BFP were 1.221([1.015 to 1.469]; *P* = 3.400 × 10^-2^) and 1.221([1.015 to 1.469]; *P* = 3.400 × 10^-2^). These estimations show a significant association between obesity characteristics and CF and SRT. Moreover, the evidence of a significant causal effect of genetically predicted BMI, BFP, WHR, and HDL on CP were 0.889([0.855 to 0.924]; *P* = 2.379 × 10^-9^), 0.846([0.798 to 0.896]; *P* = 1.440 × 10^-8^), 0.937([0.902 to 0.973]; *P* = 6.719 × 10^-4^), and 1.033([1.005 to 1.062]; *P* = 2.000 × 10^-2^). Univariable MR yielded little evidence of associations between genetically predicted adipokines and cognitive-ability markers.

**Table 2:** Univariable Mendelian randomization results.

A. Estimated causal effects of obesity, lipids, and adipokines on cognitive ability									
	BMI	BFP	WHR	HDL	LDL	TG	LEP	AgRP	ADPN
CF									
BETA	-0.204	-0.284	-0.014	0.031	0.051	-0.008	-0.019	-0.058	0.001
SE	0.035	0.052	0.04	0.03	0.151	0.028	0.013	0.144	0.05
95% CI	0.761-0.873	0.680-0.833	0.911-1.067	0.973-1.093	0.982-1.127	0.939-1.048	0.957-1.006	0.712-1.252	0.907-1.105
*P*-value	**0.000** ^b^	**0.000** ^b^	0.719	0.3	0.151	0.777	0.143	0.69	0.987
CP									
BETA	-0.118	-0.168	-0.065	0.033	-0.004	-0.019	0.027	0.028	0.003
SE	0.02	0.03	0.019	0.014	0.015	0.013	0.044	0.033	0.03
95% CI	0.855-0.924	0.798-0.896	0.902-0.973	1.005-1.062	0.967-1.026	0.957-1.006	0.942-1.120	0.963-1.098	0.947-1.063
*P*-value	**0.000** ^b^	**0.000** ^b^	**0.001** ^b^	**0.020** ^a^	0.797	0.143	0.543	0.4	0.907
SRT									
BETA	0.2	0.369	-0.042	-0.127	-0.057	0.048	0.25	-0.017	0.011
SE	0.094	0.137	0.126	0.102	0.119	0.079	0.375	0.666	0.161
95% CI	1.015-1.469	1.105-1.892	0.750-1.226	0.721-1.076	0.748-1.194	0.898-1.225	0.616-2.679	0.267-3.623	0.738-1.387
*P*-value	**0.034** ^a^	**0.007** ^b^	0.738	0.215	0.636	0.546	0.505	0.979	0.943
FIS									
BETA	-0.288	-0.346	-0.155	0.03	-0.018	-0.04	0.092	0.017	0.012
SE	0.044	0.065	0.043	0.032	0.037	0.03	0.116	0.081	0.07
95% CI	0.688-0.817	0.623-0.803	0.787-0.933	0.969-1.097	0.913-1.056	0.906-1.020	0.874-1.375	0.868-1.191	0.883-1.160
*P*-value	**0.000** ^b^	**0.000** ^b^	**0.000** ^b^	0.34	0.621	0.189	0.426	0.836	0.864
B. Estimated causal effects of cognitive ability on obesity, lipids, and adipokines									
	BMI	BFP	WHR	HDL	LDL	TG	LEP	AgRP	ADPN
CF									
BETA	-0.051	-0.025	-0.053	0.003	-0.031	-0.041	0.288	-0.039	-0.079
SE	0.027	0.021	0.055	0.03	0.029	0.048	0.09	0.154	0.064
95% CI	0.901-1.003	0.935-1.017	0.851-1.056	0.946-1.063	0.916-1.026	0.874-1.053	1.119-1.592	0.712-1.300	0.815-1.047
*P*-value	0.062	0.243	0.333	0.924	0.286	0.384	**0.001** ^b^	0.8	0.214
CP									
BETA	-0.129	-0.109	0.005	0.122	0	-0.09	-0.302	0.069	0.001
SE	0.034	0.024	0.031	0.03	0.015	0.02	0.093	0.052	0.025
95% CI	0.822-0.939	0.855-0.940	0.946-1.068	1.066-1.198	0.971-1.031	0.879-0.949	0.616-0.887	0.968-1.185	0.953-1.051
*P*-value	**0.000** ^b^	**0.000** ^b^	0.864	**0.000** ^b^	0.988	**0.000** ^b^	**0.001** ^b^	0.184	0.982
SRT									
BETA	0.004	0.005	0.009	0.001	-0.002	0.011	0.011	0.068	-0.008
SE	0.005	0.004	0.01	0.007	0.006	0.005	0.033	0.031	0.013
95% CI	0.993-1.015	0.997-1.014	0.990-1.028	0.988-1.014	0.987-1.009	1.000-1.022	0.947-1.079	1.007-1.138	0.967-1.017
*P*-value	0.464	0.203	0.356	0.907	0.727	**0.042** ^a^	0.748	**0.030** ^a^	0.521
FIS									
BETA	-0.068	-0.051	0.013	0.053	0.017	-0.03	-0.135	0.005	-0.004
SE	0.021	0.014	0.018	0.02	0.01	0.011	0.044	0.028	0.016
95% CI	0.897-0.974	0.923-0.977	0.979-1.049	1.014-1.096	0.996-1.038	0.950-0.990	0.802-0.952	0.950-1.062	0.966-1.028
*P*-value	**0.001** ^b^	**0.000** ^b^	0.453	**0.008** ^b^	0.105	**0.004** ^b^	**0.002** ^b^	0.868	0.81

a Statistically significant (*P* < 0.05).

b Statistically significant (*P* < 0.01). The bold values indicate significant differences (P < 0.05).

**Figure 5:**
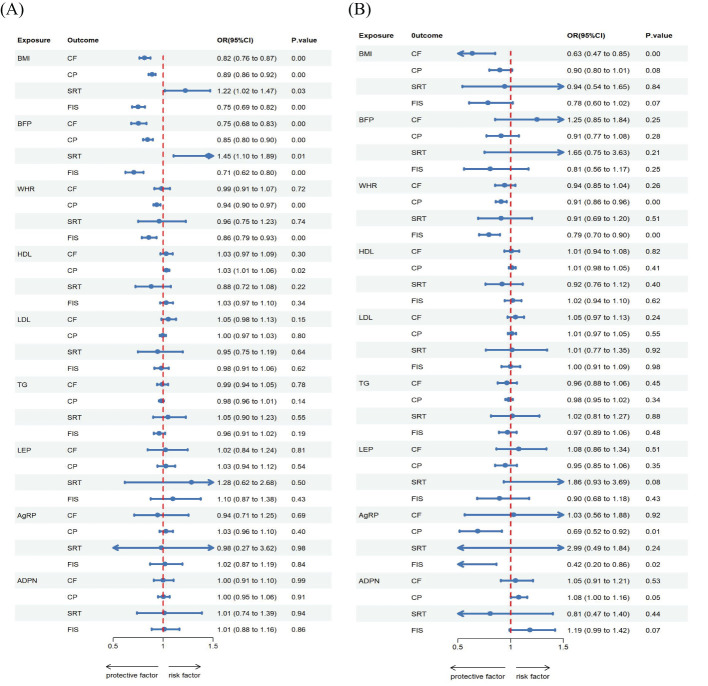
Estimated causal effects of obesity, lipids and adipokines on cognitive ability markers using both univariable and multivariable MR, presented as the SD-unit change in cognitive ability marker per genetically predicted 1-SD change in obesity, lipids and adipokines. Schematic presentation of **(A)** univariable; **(B)** multivariable Mendelian randomization.

For the reverse MR (**Table 2B**, **Figures 2C**, **3A**, **4C**, **5A**), the effect estimate ([95% CI]) of CP on BMI, BFP, HDL, TG and LEP was 0.879([0.822 to 0.939]; *P* = 1.392 × 10^-4^), 0.896([0.855 to 0.940]; *P* = 7.100 × 10^-6^), 1.130([1.066 to 1.198]; *P* = 4.058 × 10^-5^), 0.913([0.879 to 0.949]; *P* = 4.125 × 10^-6^), and 0.740([0.616 to 0.887]; p = 1.190 × 10^-3^) respectively. Furthermore, the reverse MR analysis revealed compelling evidence of a significant causal effect stemming from genetically predicted FIS on the same set of adiposity parameters. The effect estimates for FIS were as follows: 0.934([0.897 to 0.974]; *P* = 1.324 × 10^-3^) for BMI, 0.950([0.923 to 0.977]; *P* = 3.828 × 10^-4^) for BFP, 1.054([1.014 to 1.096]; *P* = 8.268 × 10^-3^) for HDL, 0.970([0.950 to 0.990]; *P* = 3.945 × 10^-3^) for TG, and 0.873([0.802 to 0.952]; *P* =2.008 × 10^-3^) for LEP. In addition, the reverse MR analysis also showed statistically significant causal effect of genetically predicted CF on LEP 1.334 ([1.119 to 1.592]; p = 1.344 × 10^-3^). There was evidence of a significant causal effect of genetically predicted SRT on TG and AG were 1.011([1.000 to 1.022]; *P* = 4.200 × 10^-2^) and 1.070([1.007 to 1.138]; *P* = 3.000 × 10^-2^).

#### Multivariable MR analyses

The MVMR analysis revealed significant causal associations between genetically predicted obesity and adipokines traits; BMI on CF, WHR on CP and FIS, AgRP on CP and FIS, ADPN on CP (**Table 3A**, **Figures 2B**, **4B**, **5B**). Specifically, BMI showed a significant negative causal effect on CF, consistent with the findings from the forward univariable analysis (β= 0.635([0.472–0.853]; *P* = 3.000 × 10^-2^). Genetically predicted WHR had a significant causal effect on CP 0.910([0.860 to 0.961]; *P* = 1.000 × 10^-2^) and FIS 0.793([0.701 to 0.900]; *P* = 0.000). Additionally, AgRP exhibited a significant negative causal effect on CF 0.687([0.515–0.916]; *P* = 1.100 × 10^-2^). In contrast, there were no significant associations between lipid traits and cognitive ability, consistent with previous analyses.

**Table 3:** Multivariable Mendelian randomization results.

A. Estimated causal effects of obesity, lipids, and adipokines on cognitive ability									
	BMI	BFP	WHR	HDL	LDL	TG	LEP	AgRP	ADPN
CF									
BETA	-0.455	0.225	-0.059	0.008	0.045	-0.037	0.073	0.029	0.047
SE	0.151	0.196	0.052	0.036	0.038	0.048	0.112	0.308	0.074
95% CI	0.472-0.853	0.854-1.838	0.851-1.044	0.940-1.081	0.970-1.128	0.877-1.060	0.864-1.340	0.563-1.884	0.906-1.213
*P*-value	**0.003** ^b^	0.25	0.257	0.82	0.241	0.45	0.511	0.925	0.526
CP									
BETA	-0.107	-0.093	-0.095	0.013	0.011	-0.017	-0.052	-0.375	0.074
SE	0.061	0.086	0.029	0.016	0.019	0.018	0.056	0.147	0.037
95% CI	0.798-1.012	0.770-1.078	0.859-0.962	0.982-1.046	0.975-1.049	0.948-1.019	0.852-1.059	0.515-0.916	1.001-1.158
*P*-value	0.078	0.279	**0.001** ^b^	0.409	0.553	0.342	0.351	**0.011** ^a^	**0.047** ^a^
SRT									
BETA	-0.057	0.501	-0.093	-0.083	0.015	0.017	0.618	1.095	-0.215
SE	0.285	0.402	0.141	0.098	0.144	0.114	0.351	0.926	0.281
95% CI	0.541-1.650	0.750-3.632	0.691-1.202	0.759-1.115	0.765-1.346	0.813-1.273	0.934-3.690	0.487-1.835	0.465-1.400
*P*-value	0.841	0.213	0.511	0.396	0.919	0.88	0.078	0.237	0.445
FIS									
BETA	-0.242	-0.215	-0.232	0.02	-0.001	-0.031	-0.111	-0.868	0.17
SE	0.133	0.189	0.063	0.039	0.045	0.044	0.139	0.368	0.093
95% CI	0.604-1.019	0.557-1.168	0.701-0.897	0.945-1.101	0.915-1.091	0.890-1.057	0.681-1.177	0.204-0.864	0.988-1.422
*P*-value	0.069	0.255	**0.000** ^b^	0.617	0.98	0.483	0.428	**0.018** ^a^	0.067
B. Estimated causal effects of cognitive ability on obesity, lipids, and adipokines									
	BMI	BFP	WHR	HDL	LDL	TG	LEP	AgRP	ADPN
CF									
BETA	-0.038	-0.028	-0.044	0.028	0.005	-0.029	0.273	0.148	-0.046
SE	0.076	0.053	0.058	0.062	0.04	0.047	0.303	0.117	0.054
95% CI	0.829-1.118	0.876-1.080	0.855-1.071	0.911-1.161	0.929-1.086	0.886-1.006	0.726-2.377	0.922-1.457	0.859-1.062
*P*-value	0.615	0.602	0.445	0.647	0.906	0.544	0.367	0.206	0.393
CP									
BETA	0.416	0.187	0.044	-0.091	-0.096	0.026	-0.947	-0.169	0.334
SE	0.218	0.153	0.178	0.177	0.114	0.136	0.503	0.331	0.152
95% CI	0.988-2.326	0.894-1.628	0.737-1.481	0.645-1.292	0.727-1.135	0.787-1.339	0.145-1.041	0.441-1.615	1.036-1.883
*P*-value	0.057	0.221	0.807	0.607	0.398	0.847	0.06	0.609	**0.028** ^a^
SRT									
BETA	0.026	0.025	-0.032	-0.051	-0.018	0.01	0.045	0.014	0.018
SE	0.023	0.016	0.018	0.019	0.012	0.014	0.052	0.034	0.016
95% CI	0.981-1.074	0.994-1.058	0.935-1.004	0.916-0.985	0.960-1.006	0.982-1.038	0.945-1.158	0.948-1.085	0.986-1.051
*P*-value	0.253	0.12	0.082	**0.006** ^b^	0.141	0.49	0.385	0.681	0.268
FIS									
BETA	-0.244	-0.133	-0.011	0.09	0.036	-0.046	0.182	0.052	-0.137
SE	0.096	0.067	0.081	0.078	0.05	0.06	0.182	0.144	0.066
95% CI	0.650-0.946	0.768-0.998	0.843-1.161	0.940-1.274	0.940-1.143	0.850-1.073	0.839-1.715	0.795-1.397	0.766-0.994
*P*-value	**0.011** ^a^	**0.047** ^a^	0.895	0.247	0.47	0.437	0.319	0.717	**0.040** ^a^

a Statistically significant (*P* < 0.05).

b Statistically significant (*P* < 0.01). The bold values indicate significant differences (P < 0.05).

The reverse MR analysis revealed significant causal association between cognitive ability and obesity, lipids and adipokines traits, as shown in the **Table 3B**, **Figures 2D**, **3B**, **4D** Specifically, for the reverse MR, the effect estimate ([95% CI]) on BMI for FIS was 0.784([0.650 to 0.946]; *P* = 1.100 × 10^-2^). The multivariable MR yielded evidence of a significant causal effect of genetically predicted SRT on HDL 0.950([0.916 to 0.985]; *P* = 6.000 × 10^-2^). However, there was no evidence of reverse causal effects from other cognitive ability traits on genetically predicted obesity, lipids, or adipokine traits in this population.

### Sensitivity analyses

We accounted for the pleiotropic effects between instrumental variables using MR-Egger. There were two instance of horizontal pleiotropy: for the effect of CP and FIS on WHR (*P* = 0.0004 and 0.018). This pleiotropic of CP on WHR effect remained (*P* = 0.0008) after removing 9 SNPs (rs112780312, rs12448902, rs148696809, rs2005078, rs2737339, rs34811474, rs4470366, rs6708515, rs78382112) which were associated with identified outliers. Furthermore, MR-PRESSO identified 6 potentially pleiotropic SNPs (rs13107325, rs34811474, rs4456117 rs4852252, rs6125540, rs7775835) of FIS on WHR effect. When we performed the analysis without these specific SNPs, there was no significant evidence of pleiotropy (*P* = 0.2780). Heterogeneity was observed in approximately half of the heterogeneity test results However, when we conducted the MR-Egger regression intercept analysis to investigate horizontal pleiotropy between the IVs, we found no significant evidence of such pleiotropy. All associations showed *P* > 0.05 for the MR-Egger intercept, indicating no substantial horizontal pleiotropy. The same pattern was observed in the reverse MR analysis. To further assess potential confounding effects due to pleiotropy between SNPs, we employed the leave-one-out approach. The results indicated no evidence of confounding caused by pleiotropy among the SNPs.

## Discussion

To the best of our knowledge, this is the first investigation that explores the potential causal relationship between obesity, lipids, adipokines and cognitive ability markers using univariable and multivariable MR approaches in both forward and reverse directions respectively. We presented evidence suggesting possible causal effects of several obesity and lipids traits, such as BMI, BFP, WHR and HDL, on cognitive ability through univariable MR analyses. However, these effects estimation altered once accounting for diverse obesity characteristics by various representative indicators using multivariable MR analyses. In the primary multivariable MR analysis conducted in the forward direction, the adipokines of AgRP and ADPN also showed evidence of a significant causal association with cognitive ability. Our reverse-MR analysis yielded compelling evidence indicating that FIS and SRT may influence BMI and HDL respectively. Collectively, these findings not only enhance our mechanistic insights into the complex interplay between obesity and cognition but also carry substantial clinical implications, underscoring potential therapeutic targets and preventive strategies for mitigating cognitive decline amidst the backdrop of obesity and dyslipidemia.

In the adiposity to cognition direction, we corroborated the direct effects of obesity and lipids on cognitive ability. The obesity and lipids traits including BMI, BFP, WHR, AgRP and ADPN have effects on cognitive ability through two primary pathways: (i) a directed path from BMI, BFP and WHR to CF, CP, SRT; (ii) a pathway from AgRP and ADPN to CP through HDL. For the causal relations between adiposity on cognition, while one study reported no significant differences in cognitive performance among normal, overweight, and obese individuals, this might be attributed to the utilization of Mini Mental State Examination (MMSE), which is not highly sensitive to mild cognitive deficits (37). Our selection of cognitive metrics was guided by their sensitivity to detect subtle changes in cognitive function and their relevance to different aspects of cognitive processing, such as memory, attention, and executive function (38, 39). Our study aligns with previous research suggesting that the obesity traits like BMI, BFP and WHR were causal factors for increasing risks of cognitive function (40, 41). Meanwhile, obesity was found to have an adverse effect on reaction speed across some epidemiological and experimental studies (42, 43), which supports our present findings. More importantly, this study was the first to reveal the effects of ADPN and AgRP on CP. An observational study observed that lower adiponectin levels in individuals with obesity (44). As the ADPN appeared to induce an increase in serum HDL (45), the protective effects of ADPN on CP might be mediated through HDL, which displayed positive associations with the ADPN, HDL, and CP in this study. Similarly, our study also found a negative association between plasma AGRP and cognitive (46), corroborating previous research. Despite previous demonstrations indicating that leptin has cognitive enhancing properties (47), our analysis did not provide evidence of a significant causal association between leptin and cognitive traits.

In the direction cognition to adiposity, although we reported that CF, CP, SRT, and FIS had a significant causal effect on obesity traits with the main univariable analysis, only SRT and FIS had a strong effect on HDL and BMI with the multivariable MR analysis. These results contradict previous studies that reported either no association or positive correlations between cognitive function and adiposity risks (8, 48). Our study, based on prospectively longitudinal data, provides robust evidence that lower cognitive function, as indicated by worse SRT and FIS, is associated with lower HDL cholesterol levels and higher BMI (49, 50). Nevertheless, it is unclear how the cognition specifically affects obesity and further studies are needed.

Furthermore, it is important to consider the broader context of obesity-related metabolic disturbances that may contribute to cognitive decline and neurodegeneration. Obesity is associated with increased levels of pro-inflammatory cytokines and chemokines, which can lead to chronic low-grade inflammation. This inflammatory state can promote neuroinflammation and gliosis, key pathological features in neurodegenerative disorders (51). Additionally, obesity-induced decreases in adipokines such as leptin, along with elevated lipid and glucose levels, may exacerbate these processes (52). Our results hint at potential causal relationships where such factors could induce gliosis and contribute to cognitive impairment, providing a mechanistic framework for the observed associations.

The strengths of our study lie in the updated genetic instruments for lipid and adipokine traits in obesity and cognitive MR analyses. We incorporated data from large consortia, which provided robust genetic evidence for the reported associations. By including TG, LEP, ADPN, and AgRP as additional traits, our study expanded the scope of genetic factors considered in the analysis. On the other hand, the utilization of data from populations of European ancestry in our MR study helped mitigate the risks of confounding and reverse causality, while minimizing bias caused by population stratification (53). Meanwhile, our study employed both univariate and multivariate MR analyses in a bidirectional design, enabling us to establish the direction of causal effects between adiposity and cognitive function.

Several limitations of our MR investigation should be acknowledged. First, we did not stratify the causal association between obesity and cognition by gender or age, which may influence fat distribution in different anatomical regions (54). Second, there are various testing methods related to cognitive function. Whether or not these tests represent directly represent cognitive related functions remained unclear. Meanwhile, the representative indicators of obesity and cognitive function included in our study are also limited. Third, the small sample size of GWAS in LEP and AgRP may resulted in imprecision in the selection of SNPs. Therefore, there is a need for larger GWAS to identify more genetic variants for adipokines. Furthermore, it is crucial to validate our findings through longitudinal cohort studies in future research.

## Conclusions

Taken together, we demonstrate that high BMI, BFP, WHR and AgRP have negative causal direct effects with cognitive ability, while high HDL and ADPN have positive causal direct effects with cognitive ability. The total effects of obesity on cognitive ability may be mediated by lipids and adipokines including HDL, ADPN and AgRP. For the reverse causal direction, there was a consistent evidence that worse cognitive function such as SRT and FIS may influence serum HDL level and BMI. These studies helped us to objectively validate previous observational studies and deepen our understanding of the association with obesity, lipids and adipokines on cognitive ability. Our study thereby contributes a pivotal piece to the clinical puzzle, elucidating potential biomarkers and pathways implicated in obesity-related cognitive decline. Nevertheless, further mechanistic explorations are imperative to decipher the precise molecular mechanisms underpinning these causal associations, which may ultimately inform targeted interventions and preventative strategies aimed at preserving cognitive integrity amidst the obesity epidemic.

## Data Availability

The raw data supporting the conclusions of this article will be made available by the authors, without undue reservation.
